# Building on the success of soil-transmitted helminth control - The future of deworming

**DOI:** 10.1371/journal.pntd.0005497

**Published:** 2017-04-20

**Authors:** Peter Mark Jourdan, Antonio Montresor, Judd L. Walson

**Affiliations:** 1 DeWorm3, Natural History Museum, London, United Kingdom; 2 Department of Control of Neglected Tropical Diseases, World Health Organization, Geneva, Switzerland; 3 Departments of Global Health, Internal Medicine (Allergy and Infectious Disease), Pediatrics and Epidemiology, University of Washington, Seattle, Washington, United States of America; Baylor College of Medicine, Texas Children's Hospital, UNITED STATES

There has been substantial progress over the past decade in efforts to reduce morbidity from soil-transmitted helminth infections (STH). Morbidity control through preventative chemotherapy (PC) has been embraced by endemic countries, the World Health Organization (WHO) and by partners as a clear and achievable goal. Few global health programs have achieved delivery of an intervention at a scale comparable to STH morbidity programs; over 4 billion tablets have been distributed to over 1 billion of the world’s most vulnerable populations [1]. Prior to the establishment of the albendazole and mebendazole donation program in 2012, global PC coverage had stagnated at approximately 30%. However, since 2015, treatment coverage has almost doubled (59%) and drug requests and donations continue to increase [2]. This progress has been particularly remarkable in the WHO South East Asia Region, home to the largest number of children in need of PC. In 2015 more than 75% of at-risk children in this region received STH PC. This remarkable global success is the result of significant collaborative efforts championed by endemic country governments, non-governmental organisations, philanthropic foundations and pharmaceutical companies [3].

In addition to achieving high PC treatment coverage, the global scale-up of STH programs has also had a substantial impact on morbidity in children [4]. Globally, PC has reduced the number of individuals with morbid STH infections (hookworm infections of any intensity, and ascariasis and trichuriasis of moderate and heavy intensity) by 85%. These data also suggest that after only one round of PC, the prevalence of STH-associated morbidity in children is reduced by approximately 75% and after 10 years of annual PC interventions, STH-associated morbidity can be virtually eliminated (WHO, 2015).

The successful scaling-up of STH PC programs and subsequent reductions in morbidity are, in part, attributable to the establishment of ambitious NTD control program goals in the WHO Roadmap [5]. However there are a number of potential challenges that may threaten the continued success of the established STH strategy. First, less than two thirds of all individuals in need of STH treatment receive PC [2]. Nearly half of those who receive albendazole are treated through the Global Program to Eliminate Lymphatic Filariasis (GPELF), not through programs designed to directly target STH. As some countries reach elimination targets and scale down their LF PC programs, STH treatment coverage may be compromised in many areas. In addition, the emergence of drug resistance to benzimidazoles (albendazole and mebendazole) has been widely reported in the veterinary literature, and as drug coverage increases, the threat to human populations is not insignificant, particularly given the lack of alternative available drug classes for treatment [6]. Finally, despite the availability of donated drugs, the financial and operational costs of PC programs are substantial and may be a barrier to endemic countries taking over the financing of STH program delivery.

These issues, coupled with uncertainty regarding the scale of drug commitments post 2020, threaten the continued success of STH programs. Sustaining progress may be particularly difficult in areas where morbidity has been reduced substantially and STH are no longer considered a public health priority. As a result, there is global interest in moving beyond control of morbidity to the elimination of STH in some geographic areas. Recent models suggest that it may be possible to interrupt STH transmission with PC alone [7,8]. In areas with moderate to low prevalence of any STH, intensive community-wide PC with albendazole appears sufficient to break transmission within a period of several years, if coverage and compliance are high (above 80%) [9].

However, given the association between access to improved water, sanitation and hygiene (WASH) and STH transmission intensity, interrupting STH transmission may not be possible without considerable improvements in access to WASH, particularly in areas where environmental contamination with human faeces is intense [10]. A recent survey conducted in the Philippines found soil-transmitted helminth eggs in 85% of soil samples [11] and helminth eggs are known to survive in the environment for many years [12]. Extensive improvements in living and sanitation standards clearly contributed to the elimination of STH in previously endemic countries, such as Japan, Italy, and Germany. As a result, the WHO recommends that STH control programs incorporate WASH activities and that the presence of NTDs be tagged as a marker of WASH access and utilisation to track progress towards the Sustainable Development Goals (SDGs). However, despite the known associations of WASH with STH transmission, it is important to note that interventional trials have not consistently demonstrated benefit in reducing STH transmission with WASH interventions of limited scale [10].

In order to determine if a strategy focusing on the elimination of STH could be pursued in some or all geographic areas, a number of issues will need to be addressed. First, to break transmission, all at-risk populations will need to be targeted. Achieving sufficiently high PC coverage of entire communities may be difficult and costly. Given the pace of current socio-economic development, a strategy targeting the global elimination of STH may be unrealistic [13]. In addition, even if interrupting transmission is possible, a strategy aimed at STH elimination is not without risk. Treatment of an entire community, including adult men, will remove an important untreated population group, or refugia, which may increase the selection of resistance mutations [6]. Moreover, given that many populations are currently treated for STH through LF programs, strategies for continued treatment of populations in areas where LF programs are scaling down are needed. Finally, elimination status can only be assessed after cessation of PC. If countries discontinue MDA programs only to find that transmission has persisted, programs may have been defunded in the interim as health priorities shift to other diseases. Reinstating programs after they have been dismantled is likely to be operationally and politically difficult and the potential failure of an elimination strategy could pose a real threat to future STH programs.

The global STH community is at a crossroads. Sustaining the incredible gains achieved by STH and LF programs will be challenging, particularly as STH prevalence and morbidity continue to decline and focus shifts to other competing global health priorities. The WHO and partners will need to continue to review the current STH strategy focused on morbidity control to ensure that STH-driven programs align with the evolving needs of STH-endemic countries, including assessing who should be treated, how frequently and the role of WASH in STH programs. While several ongoing trials aim to determine the feasibility of breaking the transmission of STH, robust evidence demonstrating that interruption of STH transmission is possible at scale does not yet exist. Elimination of STH in some geographic areas is a compelling possibility; however, it is important to continue to base global policy on available evidence until new data can support changes in strategy. The STH community must continue to advocate for and support efforts to strengthen existing programs, work with endemic governments to increase commitments to STH control, ensure continued drug donations, and to collect high-quality evidence to inform the next generation of STH programs and policies.
